# Complete Genome Sequences of Lineage IV Peste des Petits Ruminants Viruses from the Indian Subcontinent

**DOI:** 10.1128/genomeA.01009-15

**Published:** 2015-09-10

**Authors:** Aliyu Abdullahi Masdooq, Rahul Mohanchandra Pawar, Aravindh Babu R. Parthiban, K. Ragavendhar, G. Sundarapandian, A. Thangavelu, G. Dhinakar Raj

**Affiliations:** a Department of Veterinary Microbiology, Madras Veterinary College, TANUVAS, Chennai, India; b Department of Animal Biotechnology, Madras Veterinary College, TANUVAS, Chennai, India; c Translational Research Platform for Veterinary Biologicals, CAHS, TANUVAS, Chennai, India

## Abstract

The complete genome sequences of two virulent lineage IV peste des petits ruminants viruses (PPRVs) isolated from clinically infected goats in the Indian subcontinent are reported here. This is the first report of a complete genome sequence of a virulent PPRV isolate from India in recent decades.

## GENOME ANNOUNCEMENT

Peste des petits ruminants virus (PPRV), a member of the *Morbillivirus* genus of *Paramyxoviridae*, causes an acute and economically significant viral disease in small ruminants. PPRV is endemic in Asia, Africa, and the Middle East. PPRV is hypothesized to have originated in Africa (1). The disease was first reported in southern India (2) and later was detected all across the Indian subcontinent (3–7). PPRV exists as a single serotype but can be genetically classified into four different lineages, and only lineage IV of PPRV is prevalent in India (3). The molecular epidemiology of PPRV uses phylogenetic analysis of a small region of the fusion (F) gene or the nucleoprotein (N) gene (8).

Currently, at least one complete genome sequence representing each of the four lineages of PPRV is available in GenBank. For the lineage IV PPRV complete genomes, namely, those of NC_006383 from Turkey, FJ905304, JF939201, and JX217850 from Tibet, and KC594074 from Morocco, are available in GenBank. From India, only two complete genomes of PPRV (one vaccine strain Sungri [KF727981 and KJ867542] and one virulent strain [KR140086, isolated in 1994) are available. Here, we report for the first time the full-genome sequences of two virulent strains of PPRV isolated from clinically infected goats.

For the isolate (KR261605), oligonucleotide primers were designed using the conserved regions of PPRV full-length genome sequences available in the database. The primers were used to generate overlapping PCR products, which were gel purified and sequenced by Sanger dideoxy sequencing. The genome termini were determined using 3′/5′ rapid amplification of cDNA ends (RACE) (9). The complete genome sequence of the isolate (KT270355) was determined using the Illumina HiSeq sequencing platform, and the paired-end reads were aligned using Bowtie2, using KF727981 as a reference genome.

The size of the PPRV full genomes reported here are 15,948 nucleotides (nt) (KR261605) and 15,942 nt (KT270355). A six-nucleotide deletion was noticed in KT270355 in the noncoding region between the M and F genes (nucleotide positions 4487 to 4492). The genome organization was similar to that of other PPRV isolates reported from around the world. The 3′ ends of the genomes start with a genomic promoter, followed by the transcriptional units of the structural protein genes (N, P, M, F, H, and L), and end with the 5′ antigenomic promoter. Each transcription unit starts with a gene start sequence and ends with a gene end sequence. Intergenic trinucleotides were seen between the transcription units. Further availability of complete genome sequences of PPRV isolates from India will help in better understanding of the presence of host-specific lineages and the molecular evolution of this virus in India.

### Nucleotide sequence accession numbers. 

The complete genomes of the PPRVs recovered from clinically infected goats have been deposited in GenBank under accession numbers KR261605 and KT270355.
